# Study of bone marrow microstructure in healthy young adults using intravoxel incoherent motion diffusion-weighted MRI

**DOI:** 10.3389/fendo.2022.958151

**Published:** 2022-11-11

**Authors:** Wenqi Wu, Tong Gong, Jinliang Niu, Wenjin Li, Jianting Li, Xiaoli Song, Sha Cui, Wenjin Bian, Jun Wang

**Affiliations:** ^1^ Departments of Radiology, The Second Hospital, Shanxi Medical University, Taiyuan, China; ^2^ Departments of Radiology, People’s Hospital, Sichuan, China; ^3^ Department of stomatology, The Second Hospital, Shanxi Medical University, Taiyuan, China; ^4^ Department of Medical Imaging, Shanxi Medical University, Taiyuan, China

**Keywords:** marrow, intravoxel incoherent motion (IVIM), diffusion-weighted imaging, genders, microstructure

## Abstract

Bone marrow is one of the most important organs in the human body. The evaluation of bone marrow microstructure and gender-related cellular and capillary networks in healthy young adults can help to better understand the process of bone metabolism. Intravoxel incoherent motion (IVIM) provides both diffusion and perfusion quantifications without requiring intravenous contrast agent injection. In this prospective study, 60 healthy young age-matched volunteers (30 men and 30 women) underwent MRI scans at 1.5 T using multi–b-value diffusion-weighted imaging on sagittal planes covering the lumbar bone marrow. The apparent diffusion coefficient (ADC), true ADC (*D*), pseudo-ADC (D*), and perfusion fraction (*f*) were calculated from the diffusion-weighted images using the mono- and bi-exponential models. Lumbar cancellous bone (L2–L4) was selected as the region of interest. An independent *t*-test was used to detect significant differences in ADC values and IVIM parameters between men and women. The differences in IVIM parameters among the L2, L3, and L4 groups were compared with analysis of variance. The *D* and *f* values in women were significantly higher than that in men (*p* = 0.001, 0.026). However, D* was significantly lower in women than that in men (*p* = 0.001). Furthermore, there was no significant gender difference for the conventional ADC value (*p* = 0.186). Moreover, there were no significant differences in the *D*, *f*, and D* values among the L2, L3, and L4 vertebras of women or men. IVIM parameters can show differences in bone marrow between young women and men. As a non-invasive method, it can assess bone marrow microstructure, such as cellularity and perfusion.

## Introduction

The bone marrow is the fourth largest organ of the human body. The primary microstructure comprises cellular structure and blood capillary (1). The factors that regulate the content of bone marrow microstructure include development, age, and gender (2). Anemia can change the pathological state of the bone marrow microstructure, causing an increase in bone marrow cell composition and blood capillary. Malignant blood system diseases can cause bone marrow pathological hyperplasia (3). Moreover, physiological changes have been reported in disease and normal bone marrow (4). The bone composition differs between men and women. Increasing age also thins the trabecular structure of the bone, increases fat content, and reduces local perfusion, resulting in bone marrow water–fat imbalance (5, 6). Therefore, it is important to determine bone marrow values in normal populations to understand the impact of physiological factors (such as gender) on quantitative and functional parameters so that we can better characterize and assess the pathogenesis, pathophysiology, and prognosis of bone marrow diseases.

Magnetic resonance imaging (MRI) is more commonly used than other imaging modalities when evaluating marrow compositions (7). Although conventional MRI can assess the cellularity of bone marrow based on signal intensity, it does not provide a quantitative evaluation (8–10). The apparent diffusion coefficient (ADC) represents a positive correlation with the cellularity of the marrow (11) and shows a linear decrease with age (more yellow marrow and less red marrow) (12). However, the ADC value overlaps neoplastic and normal marrow (13), which is controversial when assessing vertebral body lesions (12). MR spectroscopy is widely used to quantify the marrow fat fraction (14); however, different ranges of marrow fat fraction have been reported (15, 16). Dynamic contrast-enhanced MRI (DCE-MRI) has been applied to analyze the marrow perfusion of the capillary network in patients with osteoporosis, diagnose malignant lesions, and monitor tumor treatment response (17–21). However, it is not widely used in clinical practice because of the need for intravenous injection of an MRI tracer, especially in patients with renal malfunctions.

Intravoxel incoherent motion (IVIM) is a diffusion-weighted imaging (DWI) method that uses multiple b-values and a bi-exponential signal model to calculate quantitative parameters that can separately reflect tissue microcapillary perfusion and diffusivity (22–24). The low b-values (<200 s/mm^2^), pseudoperfusion fraction (*f*), and pseudodiffusion coefficient (D*) represent the characteristics of perfusion, whereas the high b-values (200 to 1,000 s/mm^2^) and the diffusion coefficient (*D*) can reflect water diffusion that is related to tissue cellularity (25).

Gender difference is one of the factors affecting the microstructure content of bone marrow, with men and women having different performances (2, 26). The quantification of the cellular structure and blood capillary of marrow can be obtained by invasive iliac crest biopsy, which is an invasive method and subject to sampling errors (21). IVIM provides diffusion and perfusion quantification information without requiring intravenous contrast agent injection (24). It has been applied to evaluate various diseases and body organs (23, 24, 27–29) and recently for bone marrow disorders (30, 31). As a baseline study, we aim to evaluate the vertebral bone marrow microstructure of healthy young people through changes in IVIM and tissue diffusion parameters and gender differences in vertebral bone marrow cellularity and capillary perfusion capacity.

## Materials and methods

### Selection of volunteers

This cross-sectional study was approved by the ethics committee of performing site. Informed consent was obtained from all the participants included in this study. Sixty healthy volunteers were recruited: 30 men and 30 women (age range, 22 to 25 years; average age, 23 years). The inclusion criteria were as follows: (a) no lesions in the spine (e.g., spinal deformity or spinal tuberculosis); (b) no hematologic disease (e.g., leukemia, anemia, or aplastic anemia); and (c) no contraindication to MRI examination at 1.5 T. All women were in the same period of the menstrual cycle.

### Magnetic resonance imaging

MRI of the lumbar vertebral bone marrow was performed on a GE Signa 1.5-T MRI scanner (GE Healthcare, Waukesha, WI, USA) using an eight-channel MR cervical/thoracic/lumbar coil. The imaging protocol included routine fast spin echo sagittal T1-weighted (repetition time (TR), 400.0 ms; echedelaytime (TE), 9.3 ms; section thickness, 5.0 mm; spacing, 0.0 mm; number of excitation (NEX), 4; field of view (FOV), 36 × 36 cm; matrix, 320 × 192; acquisition time, 2 min 29 s). The IVIM sequence was on the basis of standard diffusion-weighted single-shot spin-echo-planar imaging with 11 b-values (b = 0, 10, 25, 50, 100, 200, 400, 600, 800, 1,000, and 1,200 s/mm^2^; TR, 2000 ms; TE, 91 ms; NEX, 4; slice thickness, 5.0 mm; FOV, 36 × 36 cm; matrix, 128 × 128; diffusion gradient directions, 3; spatial resolution, 2.8 × 2.8; acquisition time, 8 min 45 s). Fat suppression of a spectral-spatial excitation pulse was used with the IVIM sequence.

### Image analysis

The post-processing software (Functool MADC software, Advantage Windows Workstation 4.4, GE Healthcare, WI, USA) was used to analyze the IVIM parameters of vertebral marrow. The traditional ADC was calculated with the mono-exponential model using b = 0 and 600 s/mm^2^ from the multi–b-value DWI. As the main IVIM parameters, the *f*, D*, and *D* values were calculated using the bi-exponential logarithmic signal fitting equation (1), and the corresponding parameter maps were generated automatically.


(1) $$S_{b}/S_{0}=(1-f)\times e^{-bD}+f\times e^{-bD^{*}}$$

where *S_b_* is the signal intensity in different b-values, and *S*_0_ is the signal intensity when *b* is 0.

**Figure 1:**
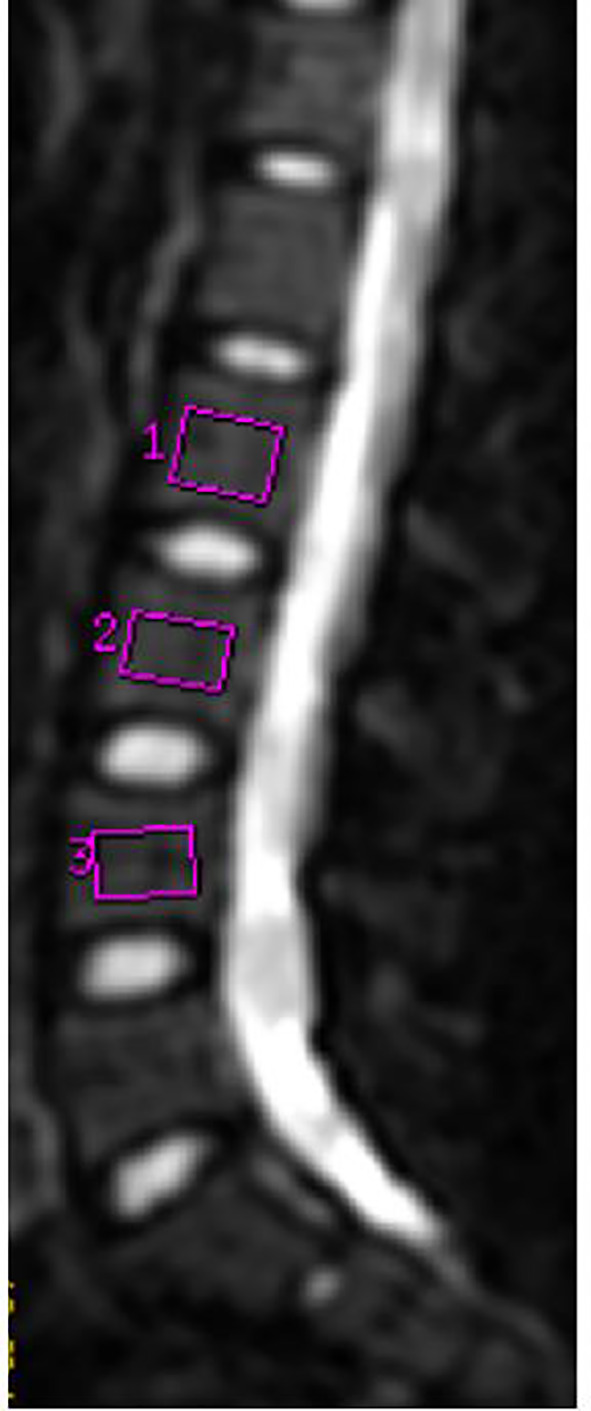
MR image in a 24-year-old woman. On the median sagittal image with b = 0, L2–L4 vertebrae were selected to delineate ROI.

The b = 0 images of the vertebral marrow, as the clearest anatomic structure among the multiple b-value images, served as a location map in the sagittal plane. The regions of interest (ROIs) were selected centrally in the lumbar vertebral cancellous bone (L2–L4) (21), which were intended to minimize the effect of vertebral end-plate changes, vertebral venous plexus, and cerebrospinal fluid. The vertebral bodies with an irregular shape, such as wedging of the vertebra, vertebrae with Schmorls node, and vertebral growth deformity, were not included in the analysis. The mean ROI size was 214 mm^2^ (range, 187–261 mm^2^) (**Figure 1**). We adopted the average value of three lumbar vertebrae (L2, L3, and L4) to compare the parameters of women and men.

### Statistical analysis

An independent *t*-test or Mann–Whitney U-test was used to detect significant differences in ADC values and IVIM parameters between men and women. Whisker plots visually describe the difference. The differences in IVIM parameters among the L2, L3, and L4 groups were compared with analysis of variance. Statistical analyses were performed using SPSS software (SPSS 19 Chicago, IL, USA). A *P*-value<0.05 was considered a significant difference.

## Results

### Comparison of parameters on women and men

**Figure 2:**
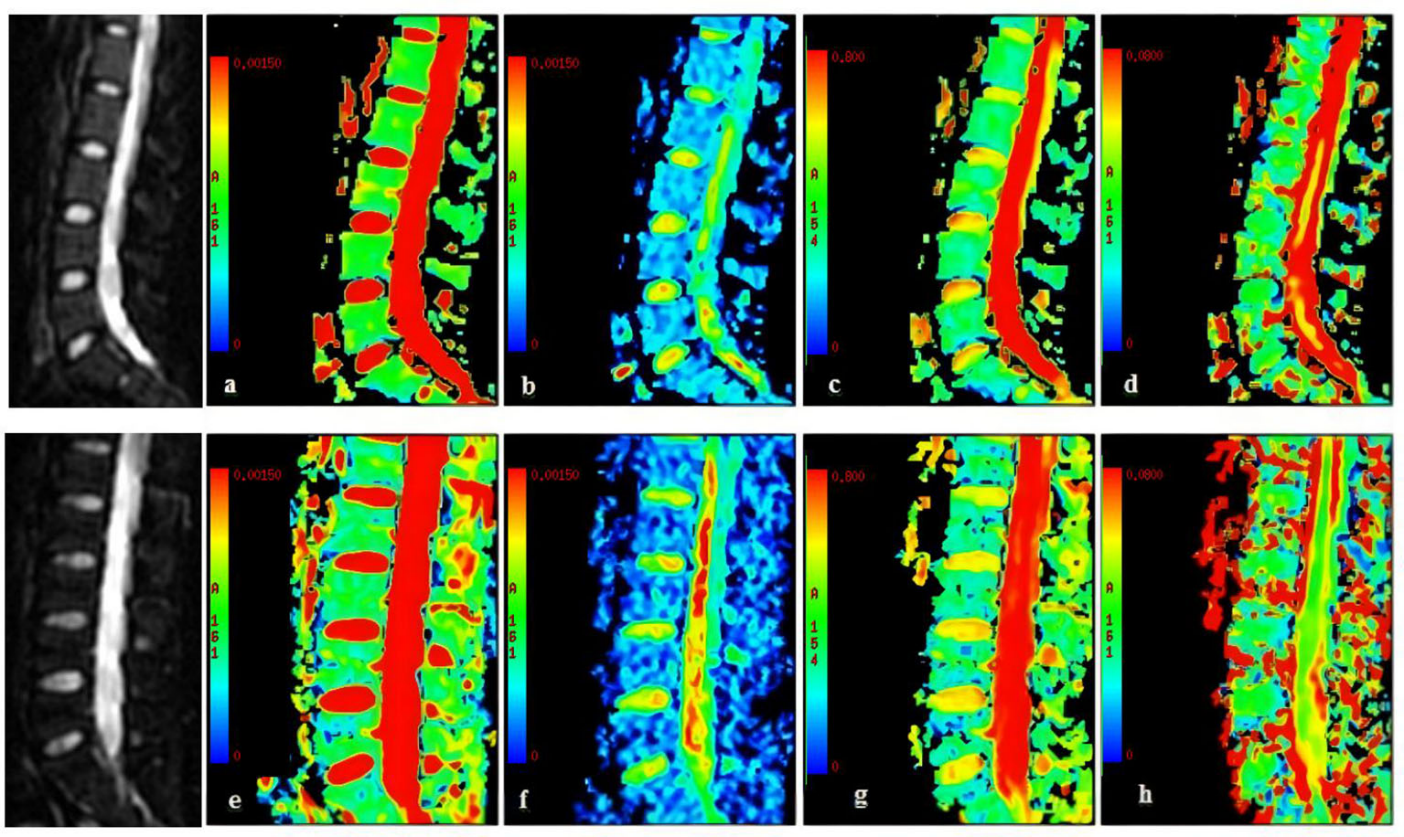
A 24-year-old woman **(A–D)**. **(A)** ADC map, ADC = 0.653 × 10^−3^ mm^2^/s; **(B)** *D* map, *D* = 0.308 × 10^−3^ mm^2^/s; **(C)** *f* map, *f* = 0.327; **(D)** D* map, D* = 46.0 × 10^−3^ mm^2^/s. A 25-year-old healthy man **(E–H)**. **(E)** ADC map, ADC = 0.588 × 10^−3^ mm^2^/s; **(F)** *D* map, *D* = 0.236 × 10^−3^ mm^2^/s; **(G)** *f* map, *f* = 0.294; **(D)** D* map, D* = 85.3 × 10^−3^ mm^2^/s. The two gray images on the left are images with b = 0.

**Figure 3:**
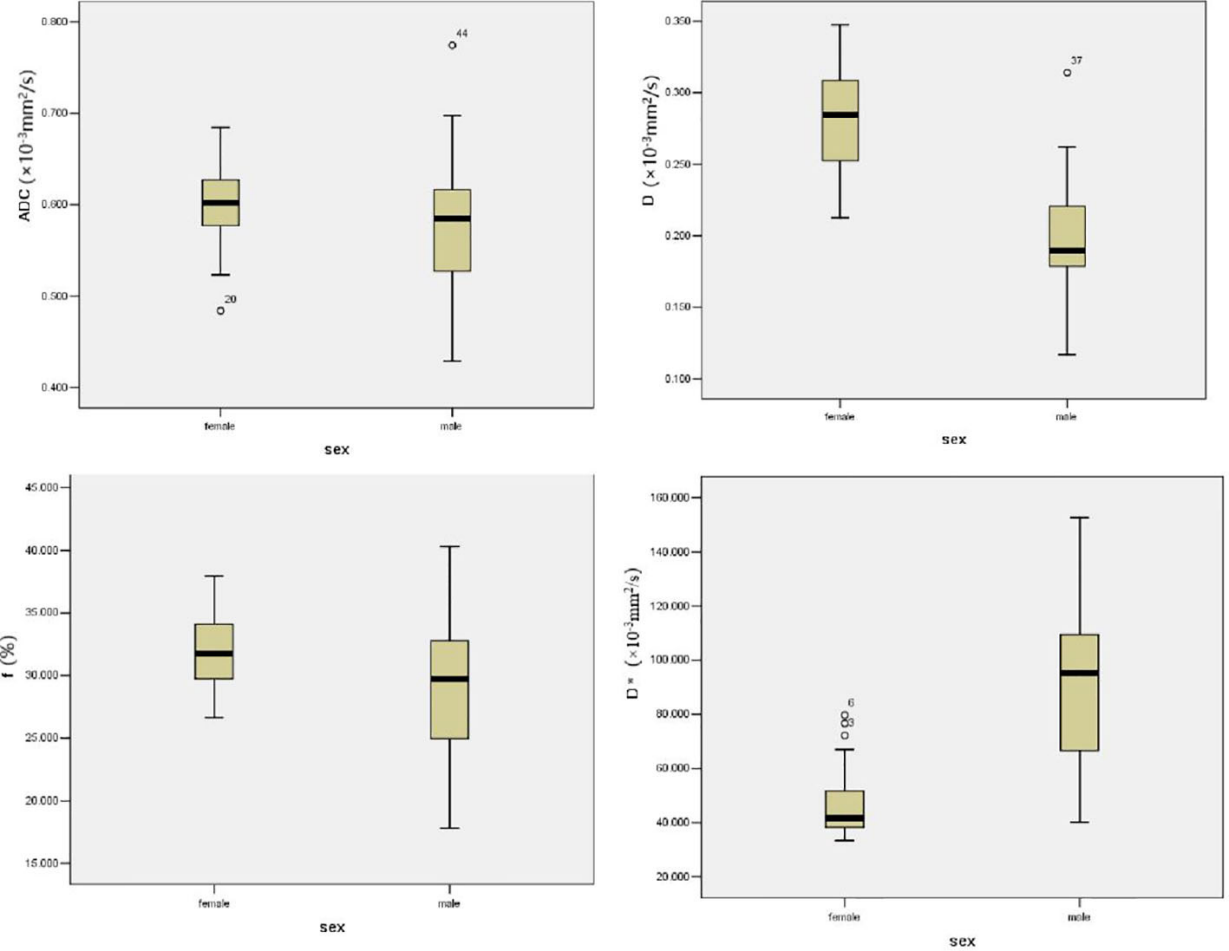
Graph shows the mean ADC, *D*, D*, and *f* values of L2–L4 for two gender groups. The horizontal bar shows the median for each group.

The ADC value of women was 0.60 ± 0.04 × 10^−3^ mm^2^/s, whereas that of men was 0.58 ± 0.07 ×10^−3^ mm^2^/s, without statistically significant differences (**Table 1** and **Figures 2**, **3**). The *D* value of IVIM, representing tissue water diffusivity, was 0.28 ± 0.04 × 10^−3^ mm^2^/s in women and significantly higher than the 0.20 ± 0.04 × 10^−3^ mm^2^/s in men (t = −8.653, *p* = 0.001; **Table 1** and **Figures 2**, **3**). In the perfusion-related parameters, *f* was significantly higher in women (31.70% ± 3.65%) compared with that in men (28.94% ± 5.52%) (t = −2.286, *p* = 0.026). However, the D* value was significantly lower in women (41.65 × 10−^3^ mm^2^/s) than that in men (95.07 × 10^−3^ mm^2^/s) (Z = −7.387, *p* = 0.001; **Table 1** and **Figures 2**, **3**).

**Table 1:** Comparison of MRI parameters of women and men.

MRI parameter	Females (n = 30)	Males (n = 30)	t/Z	p
ADC	0.60 ± 0.04	0.58 ± 0.07	−1.337	0.186
(10^−3^ mm^2^/s)	0.60 (0.57, 0.63)	0.58 (0.52, 0.63)		
*D*	0.28 ± 0.04	0.20 ± 0.04	−8.653	0.001
(10^−3^ mm^2^/s)	0.28 (0.25, 0.31)	0.19 (0.17, 0.22)		
*f* (%)	31.70 ± 3.65	28.94 ± 5.22	−2.286	0.026
	31.76 (29.71, 34.24)	29.74 (24.73, 32.93)		
D*	46.72 ± 12.90	91.78 ± 30.83	−5.544	0.001^#^
(10^−3^ mm^2^/s)	41.65 (38.09, 51.95)	95.07 (66.16, 109.69)		

Data are shown as mean ± standard deviation, mean (interquartile range). The independent t-test was applied to compare the two treatment response groups, unless otherwise specified.

^#^Determined with the Mann–Whitney U-test. Pseudodiffusion coefficient (D*) represent the characteristics of perfusion.

**Figure 4:**
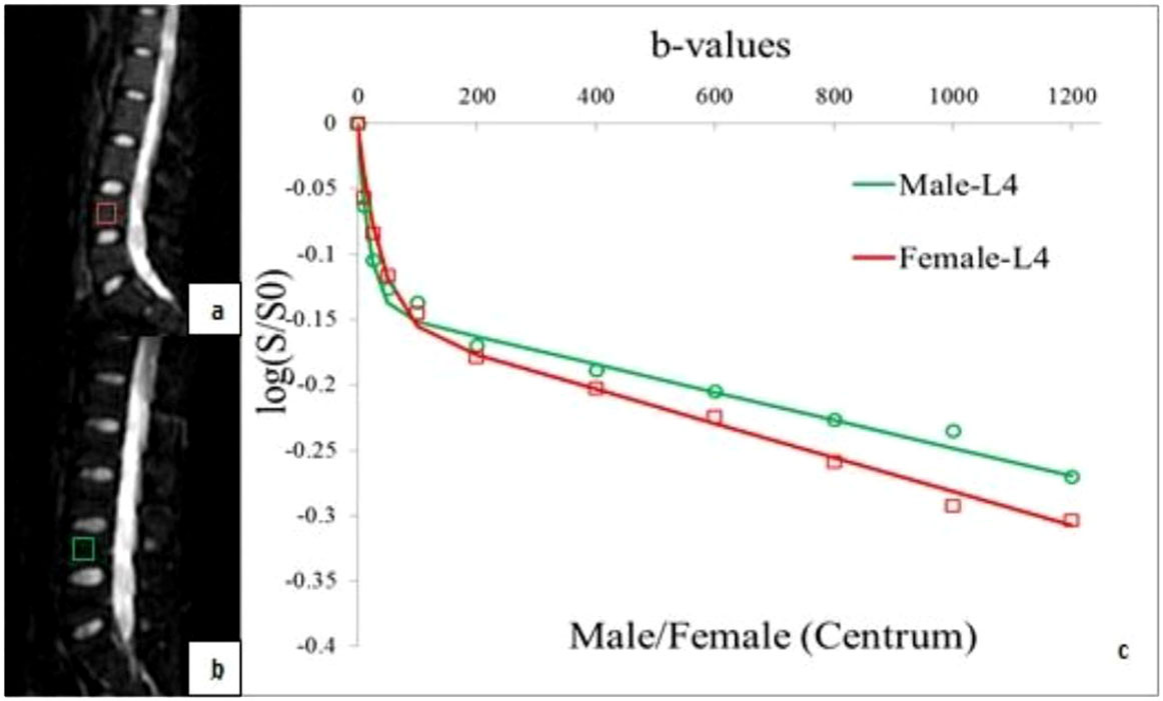
ROIs of the fourth vertebral bone marrow in a healthy 24-year-old woman **(A)** and in a healthy 24-year-old man **(B)**. The combination of their logarithmic plot of signal intensity decay and bi-exponential fitting curves **(C)**, the signal intensity decays more quickly in men than that in women within b< 200 s/mm^2^. The bi-exponential fitting curves for women (red line) and men (green line) are cross instead of parallel.

The logarithmic plot of signal intensity decay and bi-exponential fitting curves revealed differences between women and men (red and green lines, respectively), as illustrated in **Figure 4**. The ROI was in the L4 vertebral bone marrow. When b< 200 s/mm^2^, the signal intensity of men decayed faster. When b > 200 s/mm^2^, the signal decay rate of men was slower than that of women. Therefore, the bi-exponential fit curves for women and men were crossed and not parallel (**Figure 4**).

### Parameters on different vertebral segments

The *D*, *f*, and D* values were not significantly different among the L2, L3, and L4 vertebras in women (*p* = 0.978, 0.642, and 0.397, respectively; **Table 2**). Likewise, there were no significant differences in men (*p* = 0.691, 0.533, and 0.723, respectively; **Table 2**).

**Table 2:** MRI parameter of women and men in different vertebral segments.

MRI	Females (n = 30)					Males (n = 30)				
parameter	L2	L3	L4	f	p	L2	L3	L4	f	p
*D*	0.29 ± 0.05	0.28 ± 0.04	0.28 ± 0.04	0.022	0.978	0.21 ± 0.04	0.20 ± 0.04	0.20 ± 0.05	0.37	0.691
(10^−3^ mm^2^/s)										
*f* (%)	32.10 ± 3.80	30.99 ± 6.12	32.00 ± 4.95	0.445	0.642	29.13 ± 6.35	29.84 ± 6.41	27.85 ± 7.87	0.634	0.533
D*	45.98 ± 16.19	44.44 ± 8.80	49.73 ± 19.22	0.942	0.397	87.97 ± 28.32	95.03 ± 34.81	92.35 ± 38.59	0.326	0.723
(10^−3^ mm^2^/s)										

Data are shown as means ± standard deviations. L, lumbar. Determined with the analysis of variance. Pseudodiffusion coefficient (D*) represent the characteristics of perfusion.

## Discussion

Multi–b-value DWI (including a large range of b-values) reference with analysis of IVIM reflects tissue perfusion and tissue cellularity. The current study did not conduct a correlational study on bone marrow histology and IVIM in healthy volunteers. However, our previous research depicted that the *f* value was positively correlated with microvessel density in the marrow, which can be used as an alternative imaging marker of marrow angiogenesis in patients with acute leukemia and anemia (30–32). Therefore, IVIM may reflect the histology of the bone marrow in healthy people. Our current study demonstrated that the *D* and *f* values of women were significantly higher than those of men. As a non-invasive examination method, there are significant differences in IVIM parameters of bone marrow between young women and men, which provides a baseline for the evaluation of bone marrow microstructure (cellularity and perfusion).

The research on gender-related marrow composition demonstrated that women exhibited lower values of fat fraction and more cellularity compared with men of similar age (16). Although the ADC value has been correlated with the cellularity of the bone marrow (11), our study demonstrated no significant differences in the ADC values between men and women of similar ages. However, the *D* value (diffusion-related IVIM parameters) was statistically different. It suggested that the *D* value was more accurate than the ADC value in reflecting the variety of cellularity in the bone marrow. The ADC value based on a mono-exponential model was between the measured signal intensity and diffusion-weighting (b-value); however, the bone marrow was not homogeneous, and the trabeculae and fat content may cause structural tortuosity. The signal attenuation of the bone marrow was non-linear with the increasing b-value, and the ADC values included the content of tissue microcirculation perfusion (33). In previous studies, the bone marrow ADC values in young women decreased with age (34–36), but there was no significant correlation between the two in men (37, 38). The possible reason is that, with the increase in bone marrow cells in young women, the effective diffusion length is longer (37, 38).

The *f* (perfusion-related IVIM parameters) value in the bone marrow of younger women significantly increased compared with men of similar age. As the *f* value reflects the fractional volume of capillary blood flow in each voxel (25), our study depicted that the marrow perfusion and intra-medullary blood flow of younger women were higher than that of men. DCE-MRI with contrast enhancement has been useful for evaluating bone marrow perfusion (20) and has shown that the marrow perfusion of younger women (age ≤50 years) was significantly higher than that of men of similar age. This difference became less obvious in older men (age >50 years) and women (1, 39). The menstrual cycle and sex hormones of younger women were important factors for the higher bone marrow perfusion as the blood loss from menstruation may activate the hematopoietic marrow and promote red marrow (39). In our previous study, the *f* value in bone marrow was positively correlated with microvessel density, and it can be used as an alternative imaging marker of angiogenesis in the marrow in patients with acute leukemia and anemia (32). Furthermore, the *f* value can show the difference in vascularity between benign and malignant marrow disease (32). Thus, the *f* value may be a biological marker to assess the characteristics of marrow perfusion in healthy people.

The D* value was another perfusion parameter of IVIM, which was considered proportional to the mean capillary segment length and average blood velocity. A theoretical relationship between the IVIM perfusion parameter and classical perfusion parameters showed that the D* value was inversely proportional to the mean transit time (MTT) (27, 40). Our results showed that the D* value of marrow in men was significantly higher than that of women, which may suggest that the blood velocity of microcirculation in marrow was different between men and women. In men, yellow marrow is more abundant and consists of sparse capillaries, venules, and thin-walled veins. Women have more red bone marrow, which is composed of abundant dendrosinuses. The blood flow in the capillaries may be faster than the sinusoidal flow. These results suggested that the D* value could be used as an index to assess the MTT of bone marrow.

Our results indicated no significant differences in IVIM parameters among the L2, L3, and L4 vertebrae in women and men. Compared with the upper lumbar spine, the lower lumbar sustain greater stress, which may increase the pathophysiologic aging process (red to yellow marrow conversion) and decrease the perfusion inside the lower vertebral body (20, 41). DCE-MRI research reported increased perfusion of the upper lumbar spine (L2 and L3) compared with the lower (L3, L4, and L5) lumbar spine (20). The aging process of marrow alterations related to spinal level in our study was not obvious as we only investigated individuals younger than 25 years old.

We compared several previous studies using a similar approach (34, 35, 42, 43). According to the differences in DWI protocol, population, and results, we summarized the limitations of this study and areas that need to be improved in the future. First, although sex-related marrow perfusion in DCE-MRI has been reported (20), the relationship of the IVIM parameter with the microstructure of the capillary network in bone marrow can be explained if histological contrast is available, such as bone mineral density, bone markers, and sex hormone level analysis. Second, fat cells can impede water movement to a greater extent than hematopoietic cells (33). Although the red marrow was present before yellow marrow in the spine of younger volunteers (approximately 20 years old) (44), evaluating the fat fraction (FF%) could offer more information to explain our results (35). Third, limiting the study to very young volunteers reduced its clinical relevance. In the next step, we will investigate age-related bone marrow differences. Finally, compared with other IVIM parameters, the D* value map showed a low signal-to-noise ratio, especially in men. Using more b-values (0–50 s/mm^2^) may provide a more accurate estimation, whereas the decreased high b-values would improve image quality. The FOCUS-IVIM may improve its reliability (43). However, it requires high equipment and needs to be studied at 3.0 T.

## Conclusion

In conclusion, as a non-invasive imaging method, IVIM parameters can distinguish the bone marrow microstructure of young people of different gender by evaluating cellularity, vascular volume, and blood velocity of bone marrow compared with the ADC value.

## Data availability statement

The original contributions presented in the study are included in the article/supplementary material. Further inquiries can be directed to the corresponding author.

## Ethics statement

The studies involving human participants were reviewed and approved by Ethics Committee of the Second Hospital of Shanxi Medical University. The patients/participants provided their written informed consent to participate in this study.

## Author contributions

WW and TG made equal contributions to the paper and were joint first authors. All authors contributed to the article and approved the submitted version.

## Funding

This work was supported, in part, by the General project of National Natural Science Foundation of China, 82071898.

## Conflict of interest

The authors declare that the research was conducted in the absence of any commercial or financial relationships that could be construed as a potential conflict of interest.

## Publisher’s note

All claims expressed in this article are solely those of the authors and do not necessarily represent those of their affiliated organizations, or those of the publisher, the editors and the reviewers. Any product that may be evaluated in this article, or claim that may be made by its manufacturer, is not guaranteed or endorsed by the publisher.
